# Health status analysis is comparable in HM3 patients with different preoperative grades of mitral regurgitation

**DOI:** 10.1186/s41687-023-00620-9

**Published:** 2023-08-24

**Authors:** Kristin Klaeske, Constantin Schreiber, Sandra Eifert, Tillmann Dieterlen, Khalil Jawad, Diyar Saeed, Sandra Semmig-Könze, Anna Lassia Meyer, Michael Andrew Borger, Maja-Theresa Dieterlen

**Affiliations:** 1 Helios Clinic, Department of Cardiac Surgery, Leipzig Heart Center, University of Leipzig, Strümpellstraße 39, 04289 Leipzig, Germany; 2 Soteria Clinic, Clinic for Addiction Medicine, Helios Park-Klinikum, Leipzig, Germany; 3 Department of Cardiac Surgery, University Hospital Heidelberg, Heidelberg, Germany

**Keywords:** Mitral regurgitation, Short-form SF-12, Heart Mate 3, Mechanical circulatory support, Clinical psychology

## Abstract

**Background:**

The guidelines for mechanical circulatory support of the International Society for Heart and Lung Transplantation do not recommend the routine replacement or repair of the mitral valve at the time point of left ventricular assist device (LVAD) implantation. We investigated different parameters of health status including exercise capacity, anxiety and depression after LVAD implantation in patients with different preoperative grades of mitral regurgitation (MR).

**Methods:**

A single-center analysis of health status was performed including 45 patients with HeartMate 3 (HM 3) implantation using the 12-items Short Form Health Survey (SF-12) and the Hospital Anxiety and Depression Score (HADS) questionnaires. The study groups were classified according to echocardiographically defined preoperative grade of MR. The group without severe MR comprised 33 patients; the group with severe MR comprised 12 patients.

**Results:**

Demographic and preclinical characteristics as well as LVAD complications such as thrombosis and bleeding events were comparable between LVAD patients with severe and not severe MR (*p* > 0.05). Severe MR resolved in all patients after LVAD implantation and improved to moderate, mild or no MR in both groups in a period ranging from 6 months until 2 years. The analyses of SF-12 questionnaire revealed that the physical (*p* = 0.44) and mental health (*p* = 0.64) was comparable. The grade of anxiety (*p* = 0.34) and depression (*p* = 0.44) was comparable between the groups. Exercise capacity measured by the 6 min walk test correlated positively with the SF-12-determined physical health (*p* < 0.01, r = 0.518) and negatively with the HADS anxiety (*p* = 0.01, r = −0.399) and depression (*p* < 0.01, r = −0.570) scores.

**Conclusions:**

Our data showed that the health status is comparable in HM 3 patients with different preoperative MR severities in the post-LVAD period. Preoperative severe MR resolves in the majority of patients early after LVAD implantation and is not associated with concomitant mitral valve repair or replacement at the time of LVAD implantation.

**Supplementary Information:**

The online version contains supplementary material available at 10.1186/s41687-023-00620-9.

## Introduction

Functional mitral valve regurgitation (MR) is prevalent in 40–70% of the patients awaiting left ventricular assist device (LVAD) implantation. Mechanical unloading during LVAD therapy improves the mitral valve coaptation through decreased left ventricular dimension and pressure. Thus, the resolution of functional MR in LVAD patients has been detected across all grades of severity [1].

Previous reports confirmed that LVAD implantation performed without concomitant mitral valve repair may be beneficial in terms of late survival, and that pre-operative MR does not adversely affect LVAD outcomes [2–4]. The results of the interagency registry for mechanically assisted circulatory support (INTERMACS) database analysis in 2018 have shown no overall survival benefit in LVAD patients undergoing simultaneous mitral valve surgery, independent from the type of LVAD device. However, they demonstrated reduced re-hospitalization and better postoperative quality of life despite the occurrence of adverse events up to 12 months post-implant [5]. Recent data from the MOMENTUM 3 trial showed that patients with implanted magnetically-levitated centrifugal-flow HeartMate 3 (HM 3) pump had a better resolution in functional MR when compared with axial-flow HeartMate II LVAD patients [6].

Consequently, the Guidelines for mechanical circulatory support of the International Society for Heart and Lung Transplantation (ISHLT) do not recommend the routine replacement or repair of the mitral valve at the time point of LVAD implantation [7].

However, the best surgical management of secondary MR in patients evaluated for LVAD implantation is still controversial due to the disagreement of postoperative outcomes between several retrospective and prospective trials [6]. Transthoracic echocardiography data, the New York Heart Association (NYHA) classification and the 6-min walk test (6MWT) are important tools to evaluate the severity of MR and functional status of the mitral valve of LVAD patients. Nevertheless, more individualized therapeutic approaches are required to identify patients at risk for residual MR and thereby estimate the greatest benefit for those patients in terms of clinical parameters as well as health status.

Therefore, the purpose of this study was to examine the influence of different preoperative MR grades on health status of patients at the latest 2 years after HM 3 implantation and to describe the physical and mental health as well as anxiety and depression grade in post-LVAD patients in regards to MR.

## Material and methods

### Patient population

The study was conducted according to the Declaration of Helsinki, and the local ethics committee of the Medical Faculty from the University of Leipzig, Germany approved the study protocol (ID: 098/19-ek). The present retrospective observational, monocentric study included all consecutive patients with an HM 3 implantation between 2015 and 2020 at Heart Center Leipzig, Germany. Then, we excluded all patients younger than 18 years, pregnant patients, LVAD-implantations other than HM 3, patients with a mitral valve replacement due to severe structural valve defects before or simultaneously with LVAD implantation, patients with a previously implanted LVAD, missing follow-up or echocardiographic data before LVAD implantation and missing or incomplete SF-12 questionnaire. Inclusion criteria were the age of at least 18 years, HM 3 implantation and the completeness of echocardiographic evaluation and questionnaires. Depending on the grade of MR before LVAD implantation, the study cohort was divided into two groups. The group with severe MR comprised 12 patients and has been classified echocardiographically by vena contracta ≥ 7 mm, an effective regurgitation area ≥ 20 mm^2^, or a regurgitant volume ≥ 30 mL while the group without severe MR (none, mild or moderate MR) included 33 patients [8]. Severe MR was defined according to the recommendations of the European Association of Cardiovascular Imaging that differs from the recommendations of the American Society of Echocardiography (vena contracta ≥ 7 mm, an effective regurgitation area ≥ 40 mm^2^, or a regurgitant volume ≥ 60 mL). The echocardiographic evaluation was documented immediately before LVAD implantation, at the first outpatient follow up within 3 months after LVAD implantation and at the time point of health status analysis.

### Demographic and clinical data evaluation

Demographic and clinical data including the age at implantation, sex, valve surgery, etiology, implant strategy, INTERMACS profile, NYHA classification, 6MWT and echocardiographic parameters such as the left ventricular end-diastolic diameter, right ventricular end-diastolic diameter, estimated pulmonary artery pressure, tricuspid annular plane systolic excursion, tricuspid valve regurgitation were recorded. All echocardiographic data were recorded by experienced physicians.

Additionally, we collected LVAD pump parameters and post-LVAD complications such as thromboembolic, bleeding and suction events, and ventricular tachycardia.

### Instruments and measures of health status

The evaluation of the health status was performed using the SF-12 Health Survey and the Hospital Anxiety and Depression Score (HADS) in a period ranging from 6 months until 2 years following LVAD implantation.

All data were conducted during the outpatient follow-up visits using self-completed paper-based questionnaires. Both surveys are standardized and validated questionnaires, and were tested in a range of different populations with chronic disease including LVAD patients [9].

### Short-form health survey (SF-12) and short-form 6 dimension (SF-6D)

The 12-items Short-Form Health Survey (SF-12) questionnaire was designed from the SF-36 questionnaire as a shorter instrument to reflect the health status by creating a physical (PHCS) and a mental health component summary score (MHCS) [10, 11]. The SF-12 manual provides four scoring steps: (i) cleaning of out-of-range values, (ii) creating indicator variables for the item response choice categories, (iii) weighting and aggregate the indicator variables by predetermined values, and (iv) adding a standardized constant to calculate the final scores. Higher scores on PHCS and MHCS display better physical and mental health status. The possible scores for the SF-12 questionnaire range between 0 (worst mental and physical health) and 100 (best possible mental health). The scale value is representative if the patient responded to all items, otherwise the patient was excluded from the SF-12 analysis [10]. Further, the SF-12 was revised into a 6-dimensional health state classification (SF-6D [SF-12]) based on an item selection process designed by J. Brazier and J. Roberts to generate a preference-based single index. The results were transformed onto a scale with 1.0 representing full health and 0 equivalent to death [12].

### Hospital anxiety and depression scale (HADS)

The HADS questionnaire represents a well-established screening instrument that measures the presence and severity of symptoms of anxiety and depression. Both scales consist of seven items. The scores for anxiety and depression range between 0 and 21, respectively. Higher scores indicate more severe anxiety and depressive symptoms. The following cut-off values were used in this study, recommended by the authors of the HADS: scores ≥ 7.0 were considered as normal, scores ranging between 8 and 10 indicated marginal disorders and scores ≥ 11 were considered as psychically abnormal [13].

### Statistical analysis

Unless stated otherwise, the data are presented as mean ± standard deviation or as percentage proportion. Statistical analyses were performed using Intel SPSS statistical software version 28 (IBM Corp., New York, USA, 1989). Statistical significance was assigned at *p* ≤ 0.05 (two-sided). The comparison of means for demographic, clinical and psychological variables between the two study groups were executed with the Pearson’s Chi-Squared test, Fishers exact test or the Yates continuity correction in case of categorical data. Unpaired t-tests was used in the case of two-group-comparisons for normally distributed metric parameters. Mann–Whitney-U test was used in the case of two-group-comparisons for not normally distributed metric parameters.

## Results

### Patient characteristics

Demographic and baseline clinical characteristics of study patients are shown in Table 1. The age at implantation, gender, history of valve surgery, the underlying heart disease, the implant strategy, functional capacity measured by the 6MWT, and echocardiographic parameters were comparable between patients with severe and not severe MR (*p* > 0.05) (Table 1 and Additional file 1: Table S1).

**Table 1:** Demographic and baseline clinical characteristics

	Total (n = 45)	Not severe MR (n = 33)	Severe MR (n = 12)	*p* value
Age at implantation [yrs]	59.1 ± 10.7	58.2 ± 10.8	61.7 ± 10.5	0.35
Sex, male	42 (93.3%)	30 (93.3%)	12 (100.0%)	0.69
Valve surgery				
AVR during LVAD	5 (11.1%)	3 (9.1%)	2 (16.7%)	0.86
TVR during LVAD	1 (2.2%)	1 (3.0%)	0 (0%)	1
Etiology				1
NICM	22 (48.9%)	16 (48.5%)	6 (50.0%)	
ICM	23 (51.1%)	17 (51.5%)	6 (50.0%)	
Implant strategy				0.25
BTT	14 (31.1%)	10 (30.3%)	4 (33.3%)	
DT	14 (31.1%)	8 (24.2%)	6 (50.0%)	
BTD	14 (31.1%)	12 (36.4%)	2 (16.7%)	
BTR	3 (1.4%)	3 (9.1%)	0 (0%)	
INTERMACS profile				0.88
1	7 (15.6%)	5 (15.2%)	2 (16.7%)	
2	11 (24.4%)	8 (24.2%)	3 (25.0%)	
3	20 (44.4%)	14 (42.4%)	6 (50.0%)	
4–7	7 (15.6%)	6 (18.2%)	1 (8.3%)	
Pre-LVAD 6 MWT [m]	301.8 ± 95.7	306.3 ± 85.0	293.3 ± 117.7	0.72

*AVR* aortic valve replacement or reconstruction, *BTD* bridge to decision, *BTR* bridge to recovery, *BTT* bridge to transplant, *DT* destination therapy, *ICM* ischemic cardiomyopathy, *LVAD* left ventricular assist device, *INTERMACS* interagency registry for mechanically assisted circulatory support, *MR* mitral regurgitation, *MVR* mitral valve reconstruction, *NICM* non-ischemic cardiomyopathy, *TVR* tricuspidal valve replacement, *yrs* years, *6 MWT* 6-min walk test

After LVAD implantation, clinical parameters such as LVAD pump characteristics, visual assessment of right ventricular failure, 6MWT and NYHA class ≥ III were not significantly different between the two groups. Further, the frequency of suction events, thromboembolic complications such as ischemic stroke or pump thrombosis, bleeding events (cerebral bleeding, GIB, epistaxis or rethoracotomy), the incidence of ventricular tachycardia and other post-LVAD complications resulting in extracorporeal membrane oxygenation (ECMO) or RVAD implantation did not differ between the two groups (*p* > 0.05) (Table 2).

**Table 2:** Clinical characteristics and post-LVAD complications

	Total (n = 45)	Not severe MR (n = 33)	Severe MR (n = 12)	*p* value
LVAD pump parameters^†^				
Speed (rpm)	5.263 ± 222	5.292 ± 245	5.183 ± 111	0.15
Flow (L/min)	4.4 ± 0.4	4.4 ± 0.5	4.4 ± 0.4	0.96
Power (W)	3.8 ± 0.4	3.8 ± 0.3	3.9 ± 0.4	0.41
Pulsatile index	3.6 ± 0.8	3.7 ± 0.9	3.6 ± 0.6	0.72
ECMO	6 (13.3%)	3 (9.1%)	3 (25.0%)	0.37
RVAD	4 (8.9%)	2 (6.1%)	2 (16.7%)	0.61
Visual assessment RVF^‡^				0.26
Normal	2 (4.4%)	2 (6.1%)	0 (0%)	
Mildly reduced	20 (44.4%)	17 (51.5%)	3 (25.0%)	
Moderately reduced	16 (33.6%)	10 (30.3%)	6 (50.0%)	
Severely reduced	7 (15.6%)	4 (12.1%)	3 (25.0%)	
Suction events	14 (31.1%)	11 (33.3%)	3 (25.0%)	0.87
Pump thrombosis	2 (4.4%)	1 (3.0%)	1 (8.3%)	1
VT detected overall	15 (33.3%)	10 (30.3%)	5 (41.7%)	0.72
VT requiring hospital visit	5 (11.1%)	4 (12.1%)	1 (8.3%)	1
Ischemic stroke	4 (8.9%)	3 (9.1%)	1 (8.3%)	1
Bleeding events				
Cerebral bleeding	2 (4.4%)	1 (3.0%)	1 (8.3%)	1
GIB	3 (6.7%)	2 (6.1%)	1 (8.3%)	1
Epistaxis	5 (11.1%)	5 (15.2%)	0 (0%)	0.37
Re-thoracotomy	2 (4.4%)	2 (6.1%)	0 (0%)	0.96
NYHA Class ≥ III				
Post-LVAD^†^	16/39 (41.0%)	13/29 (44.8%)	3/10 (30.0%)	0.65
Time point of health status	11/40 (27.5%)	9/30 (30.0%)	2/10 (20.0%) ara>	0.70
6 MWT [m]				
Post-LVAD^†^	380.5 ± 127.1	372.4 ± 133.1	401.4 ± 113.3	0.53
Time point of health status	431.4 ± 98.1	431.2 ± 95.9	431.8 ± 107.2	0.99

Irrespectively of the number of complication episodes, the number of patients with the specific complication was counted

*ECMO* extracorporeal membrane oxygenation, *GIB* gastrointestinal bleeding, *LVAD* left ventricular assist device, *MR* mitral regurgitation, *NYHA* New York Heart Association, *RVF* right heart failure, *RVAD* right ventricular assist device, *VT* ventricular tachycardia, *6 MWT* 6-min walk test, all adverse events were defined as stated in “INTERMACS Adverse Event Definitions: Adult and Pediatric patients “ (May 15, 2013)

^†^Measured/documented at the first outpatient follow-up within 3 months after LVAD implantation

^‡^Visual assessment of RVF is a qualitative assessment of RVF by the echocardiographer

**Fig. 1:**
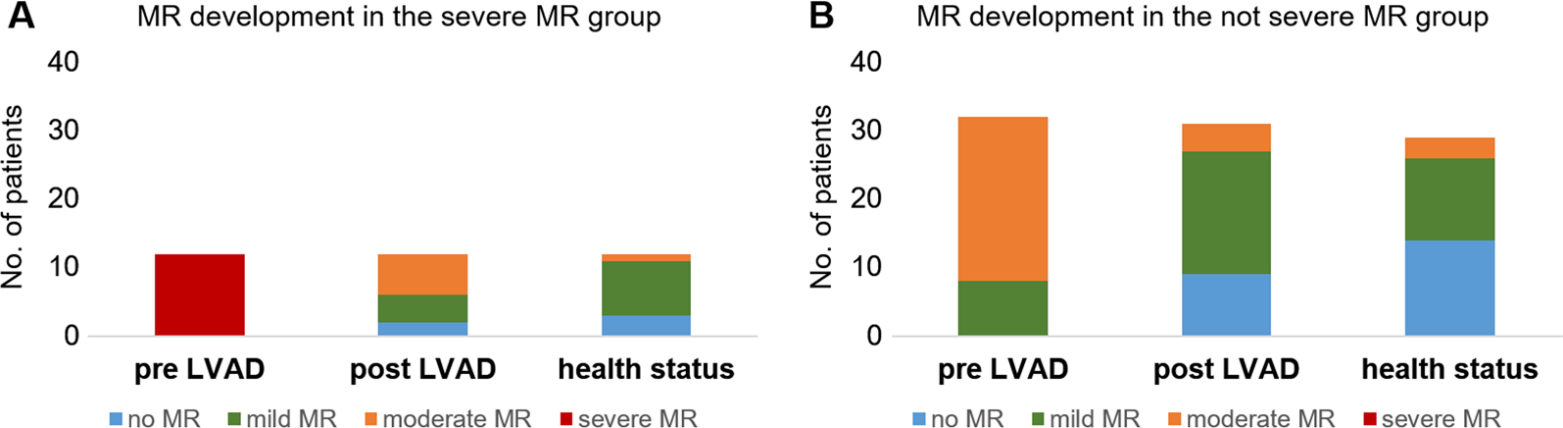
Mitral valve regurgitation development in patients with severe (**A**) and not severe MR (**B**) prior LVAD implantation and in the follow-up period. The follow-up included the post LVAD period within the first 3 months after LVAD implantation and the time point of health status analysis. MR, mitral regurgitation; post, within the first 3 months after LVAD implantation; pre, prior to LVAD implantation

The development of MR and their absolute frequencies were monitored in the group with severe MR and not severe MR prior LVAD implantation, in the post LVAD period within the first 3 months after LVAD implantation and at the time point of health status analysis. Severe MR resolved in all patients immediately after LVAD implantation to moderate (n = 6), mild (n = 4) or no MR (n = 2). At the time of health status analysis, most patients of the severe MR group had mild MR (67%) or no MR (25%). Patients in the not severe MR group, had moderate (n = 25) or mild (n = 8) MR before LVAD implantation, which improved in most patients to mild MR (56%) in the post LVAD period and no MR (45%) at health status analysis. (Table 3, Fig. 1A).

**Table 3:** MR development in the not severe MR group and severe MR group

	Not severe MR (n = 33)	Severe MR (n = 12)
Pre LVAD		
Severe MR	0 (0%)	12 (100%)
Moderate MR	25 (76%)	0 (0%)
Mild MR	8 (24%)	0 (0%)
No MR	0 (0%)	0 (0%)
Post LVAD*		
Severe MR	0 (0%)	0 (0%)
Moderate MR	5 (16%)	6 (50%)
Mild MR	18 (56%)	4 (33%)
No MR	9 (28%)	2 (17%)
Health status^$^		
Severe MR	0 (0%)	0 (0%)
Moderate MR	3 (10%)	1 (8%)
Mild MR	14 (45%)	8 (67%)
No MR	14 (45%)	3 (25%)

*MR* mitral regurgitation, *pre* prior to LVAD implantation; post LVAD, within the first 3 months after LVAD implantation; health status, and the time point of health status analysis

*One patient of the not severe group was missing

^$^Two patients of the not severe group were missing

### Questionnaires

#### SF-12 and SF-6D analysis

Seven patients (n = 2 in the severe group, n = 5 in the not severe group) did not respond to all items of the questionnaire and were excluded from the SF-12 analysis. The analysis of the SF-12 questionnaire revealed that the PHCS (not severe MR: 39.9 ± 9.0, severe MR: 37.4 ± 8.8; *p* = 0.44) and the MHCS (not severe MR: 52.8 ± 10.9, severe MR: 51.7 ± 9.7; *p* = 0.64) were comparable between LVAD patients with severe and not severe MR. The PHCS (*p* < 0.01, r = 0.524), but not the MHCS (*p* = 0.22), positively correlated with the physical activity measured by the 6MWT. The average time of evaluated SF-12 values was 14.3 ± 5.3 months.

Table 4 represents the SF-12 sub-scales for both study groups. A significant reduction of SF-12 vitality (*p* = 0.02) was observed in patients with severe MR compared to not severe MR patients.

**Table 4:** SF-12 sub-analysis

	Total (n = 45)^†^	Not severe MR (n = 33)^†^	Severe MR (n = 12)^†^	*p* value
SF-12 physical functioning	49.9 ± 3.8	50.2 ± 3.7	49.2 ± 4.0	0.49
SF-12 role-physical	51.3 ± 4.8	51.5 ± 4.9	51.0 ± 4.8	0.81
SF-12 bodily pain	52.5 ± 2.8	52.5 ± 3.0	52.6 ± 2.5	0.96
SF-12 general health	53.2 ± 1.4	53.3 ± 1.4	53.0 ± 1.4	0.57
SF-12 vitality	58.0 ± 1.8	58.5 ± 1.8	57.0 ± 1.4	0.02
SF-12 social functioning	58.3 ± 2.9	58.5 ± 2.5	57.9 ± 2.5	0.51
SF-12 role-emotional	57.0 ± 5.7	56.3 ± 6.0	58.5 ± 5.1	0.27
SF-12 mental health	55.5 ± 4.1	55.9 ± 4.3	54.4 ± 3.7	0.32

^†^Seven patients (n = 2 in the severe group, n = 5 in the non-severe group) did not respond to all items of the questionnaire and were excluded from the SF-12 analysis; MR, mitral regurgitation; SF-12, 12-items short-form health survey

The calculation of SF-6D utility scores revealed no significant difference between severe MR (0.67 ± 0.13) and not severe MR patients (0.68 ± 0.14, *p* = 0.76). The SF-6D response rate (89%) was slightly higher than for SF-12 subscales (84%), but 5 patients (n = 2 in the severe group, n = 3 in the not severe group) still had to be excluded from the SF-6D analysis.

#### HADS analysis

Four patients (n = 1 in the severe group, n = 3 in the not severe group) did not complete the questionnaire (anxiety-related, depression-related or both). Psychosocial outcomes in terms of symptoms of anxiety and depression revealed no significant differences between LVAD patients with severe MR (anxiety score: 5 ± 3; depression grade: 6 ± 3) compared to patients with not severe MR (anxiety score: 6 ± 4, *p* = 0.34; depression grade: 5 ± 4, *p* = 0.44). The frequency of patients with higher, physically abnormal scores of anxiety (severe MR: 8%, not severe MR: 9% *p* = 1) and depression (severe MR: 8%, not severe MR: 9%, *p* = 1) were comparable between the severe and not severe MR group. The grade of anxiety and depression measured by the HADS questionnaire negatively correlated with the physical activity determined by the 6MWT (anxiety: *p* = 0.01, r = −0.399; depression: *p* < 0.01, r = −0.570). The average time of evaluated HADS values was 13.8 ± 5.1 months.

## Discussion

Secondary functional MR resolves in the majority of patients undergoing LVAD implantation [1–4]. The therapeutic approaches orientate on the clinical outcomes and the greatest benefit for the patients, which includes health status analysis. Therefore, this study analyze health status parameters in patients with different pre-LVAD MR grades.

We suggest that the worse health status of patients with preoperative severe MR compared with non-severe MR may indicate that concomitant MV repair may be of additional benefit to patients with severe MR. Comparability of groups would in turn strengthen the ISHLT recommendation. The principal finding in our small study cohort indicates that PHCS, MHCS and SF-6D utility scores as well as the HADS anxiety and the depression grades are comparable in HM 3 patients with preoperative severe and not severe MR.

Secondary mitral regurgitation remains a serious entity in LVAD patients. The majority of previous studies demonstrated that LVAD therapy by itself reduced the pathology of mitral valve regurgitation regardless of preoperative MR severity because of mechanical unloading of the left heart ventricle [1–3, 6, 14]. Therefore, the guidelines for mechanical circulatory support of the ISHLT do not recommend the routine replacement or repair of the mitral valve at the time point of LVAD implantation [7]. In our study, we also observed that preoperative severe MR resolved in all patients after LVAD implantation and improved to moderate, mild or no MR in the long-term of both groups. In addition, the MOMENTUM 3 trial demonstrated an improvement in the severity of uncorrected MR within 1 month after HM 3 implantation [6].

However, the goal of an effective LVAD therapy includes, in addition to clinical parameters, the psychological assessment of emotional distress and coping strategies prior to LVAD implantation and should be integrated into outpatient care for long-term device management, especially in the context to the indication of the device, goals and intent of treatment [15]. Robertson et al. showed that patients with surgical treatment of functional MR had an improved functional status and quality of life compared to patients without MV procedure. In addition, these patients exhibited a lower incidence of hospital admissions and late heart failure-related causes [5].

In this study, we used various instruments to capture different aspects of patients’ health status. The SF-12 questionnaire was used to score the impact of general health on daily living of LVAD patients without showing a difference in physical and mental health between the study groups. The SF-12 survey was developed from the SF-36 as a shorter instrument to assess the health status in heart failure patients [16]. Previous studies demonstrated that both, the SF-12 and SF-36 scales, were comparable in validity and sensitivity to record the current health status [17]. Because of the shorter form, the SF-12 questionnaire is more practicable in the clinical and outpatient routine, and most individuals completed the SF-12 in less than 2 min without assistance, which saves time and resources. The results of our SF-12 sub-analysis showed that the scoring parameter of vitality was significantly reduced in patients with severe MR compared to patients with not severe MR. However, with regard to the results of our cross-sectional study, it is not entirely clear whether severe MR affects vitality or if the reduced quality of life has an impact on health outcomes.

The mean utility scores of SF-6D reported in our study did not differ in patients with and without severe MR, but these preference-based single indices could be used in economic evaluation after LVAD implantation and provide information on the cost-utility of this intervention [12].

The 6MWT was used as an additional parameter to assess the physical function in LVAD patients and complemented the health-related category of the SF-12 questionnaire [18]. Furthermore, a positive correlation between the 6MWT and the PHCS was documented, reinforcing the results of the SF-12 physical health scoring. The regular measurements of the 6MWT showed an improvement of physical activity after LVAD implantation in general, but no difference between patients with severe or not severe MR.

We also reported the psychological evaluation of anxiety and depression symptoms in LVAD patients with different pre-LVAD MR severities. In our study, 40% of the LVAD patients suffered from moderate to strong symptoms of depression and anxiety respectively, regardless of MR severity. For patients with severe and not severe pre-LVAD MR similar anxiety and depression grades could be documented. A negative correlation was calculated for the physical functioning measured by the 6MWT distance and anxiety and depression grades. This finding is in accordance with studies reporting a relationship between the exercise capacity measured with the 6MWT and anxiety and depression [19, 20].

This study is limited by its monocentric design and the small sample size. The results cannot be generalized. A further limitation is the non-standardized time point for SF-12 and HADS assessment and the absence of pre-surgery health status scores, which may represent a potential bias in the interpretation of study findings. Bias could be caused by individual differences in the adaptation to LVAD handling or administrated medication including anticoagulation in the short- and long-term follow-up period. In addition, due to missing data, not all patients could be evaluated in the SF-12 analysis. A larger patient cohort is required to increase the study power, verify our initial results and further evaluate if the degree of pre-implant MR would be associated with LVAD outcomes that would affect quality of life. In future prospective studies, the comparison of psychological factors before LVAD implantation and at standardized time points postoperatively in patients with and without severe MR after LVAD implantation could improve the assessment of health status in these patients.

## Conclusion

In conclusion, this study of HM 3 patients with different preoperative MR severity and without MR surgery during LVAD implantation documented a comparable health status in the post-LVAD period in a small study cohort. Further, preoperative severe MR resolves in the majority of patients early after LVAD implantation and is not associated with concomitant mitral valve repair or replacement at the time of LVAD implantation. However, as the number of implanted LVADs increases, the assessment of psychological factors and health status to clinical outcomes becomes more important and should be considered in future research and clinical practice.

### Supplementary Information

**Additional file 1**. Echocardiographic parameters.

## Data Availability

The datasets used and/or analyzed during the current study are available from the corresponding author on reasonable request.

## Abbreviations

ECMO: Extracorporeal membrane oxygenation
HM 3: HeartMate 3
HADS: Hospital Anxiety and Depression Score
INTERMACS: Interagency registry for mechanically assisted circulatory support
ISHLT: International Society for Heart and Lung Transplantation
LVAD: Left ventricular assist device
MHCS: Mental health component summary score
MR: Mitral valve regurgitation
NYHA: New York Heart Association
PHCS: Physical health component summary score
SF-12: 12-Items Short-Form Health Survey
6MWT: 6-Minute walk test
