# Supplementary material for: Health status analysis is comparable in HM3 patients with different preoperative grades of mitral regurgitation
Source: J Patient Rep Outcomes. 2023 Aug 24;7:86. doi: 10.1186/s41687-023-00620-9 (PMC10449706; doi:10.1186/s41687-023-00620-9)
Supplement: Supplementary file 1 — Additional file 1. Echocardiographic parameters. [file 41687_2023_620_MOESM1_ESM.docx]

**Supplemental Table 1: Echocardiographic parameters.**

|  | total  (n = 45) | not severe MR  (n = 33) | severe MR  (n = 12) | p value |
| --- | --- | --- | --- | --- |
| prior LVAD implantation |  |  |  |  |
| LVDD [mm] | 72.8 ± 8.0 | 72.1 ± 8.3 | 74.4 ± 7.5 | 0.20 |
| RVDD [mm] | 46.2 ± 8.3 | 45.1 ± 9.3 | 49.3 ± 3.5 | 0.02 |
| TAPSE [mm] | 15.2 ± 4.2 | 15.6 ± 4.5 | 14.0 ± 3.2 | 0.15 |
| PAPs [mmHg] | 40.5 ± 13.1 | 37.9 ± 13.7 | 46.4 ± 10.0 | 0.04 |
| TR ≥3 | 11/45 (24.4%) | 7/33 (21.2%) | 4/12 (33.3%) | 0.66 |
| post-LVAD implantation^†^ |  |  |  |  |
| LVDD [mm] | 63.0 ± 9.6 | 61.8 ± 9.3 | 66.2 ± 10.1 | 0.19 |
| RVDD [mm] | 44.4 ± 6.1 | 44.5 ± 6.5 | 44.0 ± 5.3 | 0.40 |
| TAPSE [mm] | 11.9 ± 2.8 | 12.0 ± 2.7 | 11.5 ± 3.0 | 0.32 |
| PAPs [mmHg] | 24.0 ± 8.6 | 23.3 ± 7.7 | 25.1 ± 10.3 | 0.30 |
| TR ≥3 | 2/45 (4.4%) | 1/33 (3.0%) | 1/12 (8.3%) | 1 |
| time point of health status analysis |  |  |  |  |
| LVDD [mm] | 63.7 ± 12.0 | 62.4 ± 11.5 | 67.1 ± 13.2 | 0.25 |
| RVDD [mm] | 42.3 ± 5.6 | 41.9 ± 6.0 | 43.4 ± 4.5 | 0.21 |
| TAPSE [mm] | 12.0 ± 3.3 | 12.4 ± 3.6 | 11.0 ± 2.3 | 0.12 |
| PAPs [mmHg] | 23.3 ± 7.4 | 22.2 ± 6.6 | 26.1 ± 8.7 | 0.10 |
| TR ≥3 | 3/42 (7.1%) | 2/30 (6.7%) | 1/12 (8.3%) | 1 |

Footnote Supplemental Table 1: ^†^ measured/documented at the first outpatient follow-up within 3 months after LVAD implantation; LVAD left ventricular assist device; LVDD, left ventricular end-diastolic diameter; MR, mitral regurgitation; PAPs, pulmonary artery systolic pressure; RVDD, right ventricular end-diastolic diameter; TAPSE, tricuspid annular plane systolic excursion; TR, tricuspid regurgitation
